# Uncovering the Magnetic Particle Imaging and Magnetic Resonance Imaging Features of Iron Oxide Nanocube Clusters

**DOI:** 10.3390/nano11010062

**Published:** 2020-12-29

**Authors:** Sahitya Kumar Avugadda, Sameera Wickramasinghe, Dina Niculaes, Minseon Ju, Aidin Lak, Niccolò Silvestri, Simone Nitti, Ipsita Roy, Anna Cristina S. Samia, Teresa Pellegrino

**Affiliations:** 1 Istituto Italiano di Tecnologia, via Morego 30, 16163 Genoa, Italy; sahitya.avugadda@iit.it (S.K.A.); dina.niculaes@gmail.com (D.N.); lak.aidin@physik.uni-muenchen.de (A.L.); niccolo.silvestri@iit.it (N.S.); simone.nitti@iit.it (S.N.); 2 Department of Chemistry, Case Western Reserve University, 10900 Euclid Avenue, Cleveland, OH 44106, USA; smw151@case.edu (S.W.); mxj250@case.edu (M.J.); 3 Department of Materials Science and Engineering, University of Sheffield, Sheffield S10 2 TN, UK; i.roy@sheffield.ac.uk

**Keywords:** iron oxide nanocubes, nanoclusters, magnetic particle imaging, magnetic resonance imaging, enzymatic responsive materials, 2D-Clusters, nanochains, magnetic beads

## Abstract

Multifunctional imaging nanoprobes continue to garner strong interest for their great potential in the detection and monitoring of cancer. In this study, we investigate a series of spatially arranged iron oxide nanocube-based clusters (i.e., chain-like dimer/trimer, centrosymmetric clusters, and enzymatically cleavable two-dimensional clusters) as magnetic particle imaging and magnetic resonance imaging probes. Our findings demonstrate that the short nanocube chain assemblies exhibit remarkable magnetic particle imaging signal enhancement with respect to the individually dispersed or the centrosymmetric cluster analogues. This result can be attributed to the beneficial uniaxial magnetic dipolar coupling occurring in the chain-like nanocube assembly. Moreover, we could effectively synthesize enzymatically cleavable two-dimensional nanocube clusters, which upon exposure to a lytic enzyme, exhibit a progressive increase in magnetic particle imaging signal at well-defined incubation time points. The increase in magnetic particle imaging signal can be used to trace the disassembly of the large planar clusters into smaller nanocube chains by enzymatic polymer degradation. These studies demonstrate that chain-like assemblies of iron oxide nanocubes offer the best spatial arrangement to improve magnetic particle imaging signals. In addition, the nanocube clusters synthesized in this study also show remarkable transverse magnetic resonance imaging relaxation signals. These nanoprobes, previously showcased for their outstanding heat performance in magnetic hyperthermia applications, have great potential as dual imaging probes and could be employed to improve the tumor thermo-therapeutic efficacy, while offering a readable magnetic signal for image mapping of material disassemblies at tumor sites.

## 1. Introduction

Early detection and monitoring of biomarkers associated with cancer progression is a key factor in the effective diagnosis and treatment of the disease. Different detection methods, based on the recognition of cell surface cancer biomarkers or predictive biomarkers in the tumor microenvironment, have been developed, including enzyme-linked immunosorbent assay (ELISA), gel electrophoresis, polymerase chain reaction (PCR), electrochemical, magnetic relaxometry, and fluorescence-based methods [1,2,3]. However, to date, there remains a growing interest in the use of nanoparticle-based platforms not only for cancer screening but also as therapeutic tools and at the same time, as a complementary tool to improve diagnostic imaging methods and technologies. Among such technologies, prominent ones are magnetic particle imaging (MPI) and magnetic resonance imaging (MRI). MPI is an emerging imaging technique that allows the direct quantitative mapping of spatially distributed magnetic nanoparticles (MNPs) with increased sensitivity and short image acquisition times [4,5]. Moreover, in MRI, the MNPs such as superparamagnetic nanoparticles or their corresponding assemblies, are also widely employed as contrast agents as they create dishomogeneous fields, which affects the spin-spin (*T*_2_) and spin-lattice (*T*_1_) relaxation of protons in tissues, providing a darker or a brighter signal contrast where they accumulate, respectively [6]. For detailed anatomical or structural information, tomographic modalities such as MRI or computer tomography (CT) is employed to co-register the MPI signal with anatomical information [7]. As such, adaptation of the dual MPI/MRI imaging approach, both based on the use of MNPs as contrast agents, can raise the diagnostic potential of the individual magnetic imaging modalities by combining complementary information [8]. For instance, Kaul et al. in 2015 presented the first preclinical study in a mice model that established a workflow of combining MPI and MRI [9]. In another study, Salamon, J. et al. evaluated a real-time 4D tracking of endovascular devices and stenosis treatment by merging MPI and MRI imaging modalities in an in vitro model [10].

In MPI, the signal derives from the non-linear magnetization of iron oxide-based nanoparticles used as tracer. One established MPI technique is based on an x-space reconstruction approach that was developed by Conolly’s group, where the image is expressed as a convolution of the MNPs spatial distribution with the point spread function (PSF) of the system [5]. Image resolution in x-space MPI is determined by the full-width at half-maximum (FWHM) of the PSF. To enable the fast screening of MPI tracers, a magnetic particle imaging relaxometer was developed [2,11,12,13]. To excite the MNP tracer, a sinusoidal wave with frequencies typically in the 15–25 kHz range is generated in the transmit coil, which subsequently results in a temporal change in the magnetization of MNPs that is detected in the receive coil. The signal derived from MPI relaxometry measurements is then used to characterize the magnetization dynamics of the MNP tracers as well as their average concentration and size distribution [2,4,5,13,14].

Moreover, MPI is not only a sole imaging modality, it is also becoming an auxiliary tool for magnetic hyperthermia (MH) [15,16]. This combined diagnostic and therapeutic approach is doable because the way MNPs are excited in MPI is similar to that in MH, where alternating magnetic fields are used. However, the range of frequency required for MPI differs from that of MH (*f* ≤ 35 kHz versus *f* ≥ 110 kHz, respectively) [17]. Despite the use of a clinically approved MRI contrast agent, Resovist^®^, as a common standard, the low heat performance of Resovist^®^ and the current availability of a wide portfolio of MNPs, has pushed early research towards the exploitation of optimal MNPs with combined MPI, MRI, and MH properties [18]. To mention an example, among the MNPs recently available, magnetosomes, which are MNPs produced by magnetotactic bacteria, exhibit the best MPI efficiency [19] coupled with outstanding MH heat performance and MRI properties [20,21]. Moreover, while MNPs organized in different structural assemblies have been previously investigated with respect to their MH heat performance [22,23], the correlation of the structure of the MNP assemblies to the MPI signals has not been investigated to date. It is also important to note that as with the MH and MRI performance, the effective MPI relaxation of MNPs depends on several factors such as the magnetic core size, magnetic properties, anisotropy [13], dipole interactions/alignment of the easy axis, and their alignment to external fields that can significantly affect the MPI signal [17].

In this study, we carried out a dual MPI and MRI study on different clusters of iron oxide nanocubes with well-defined geometries. These clusters were previously reported for their MH heat efficiencies and included chain-like assemblies, centrosymmetric clusters (3D-Clusters) [24] and two-dimensional clusters (2D-Clusters); [25] the latter having peculiar disassembly property that is promoted by esterase enzymatic action on aliphatic polyester (polyhydroxyalkanoates) polymers used for the clustering of iron oxide nanocubes (IONCs), which can undergo enzymatic cleavage into short nanocube chains upon esterase exposure [25]. This enzyme is abundant in the tumor microenvironment [26] and can cleave polyester polymers like the one used to encapsulate the 2D-Clusters. In this study, we identified the chain-like assemblies as the most efficient MPI tracers among the various clusters investigated. In addition, we demonstrated the possibility to employ the 2D-Clusters as potential MPI and MRI probes to identify and monitor lytic enzyme activity under simulated tumor microenvironment conditions.

## 2. Materials

Poly(styrene-co-maleic anhydride) cumene-terminated (M_n_ = 1600 g/mol), tetrahydrofuran (THF, 99%), chloroform (CHCl_3_, 99%), 1-octadecene (1-ODE, 99%), iron pentacarbonyl (Fe(CO)_5_, 98%), oleic acid (OLAC, 90%), and liver porcine esterase were purchased from Merck Life Science S.r.l. (Milan, Italy). Iron(III) acetylacetonate (99%), decanoic acid (99%), and dibenzyl ether (99%) were purchased from Acros Organic and distributed by Fisher Scientific Italia (Milan, Italy). Squalane (98%) and sodium oleate (97%) were from Alfa Aesar (Kandel, Germany) and TCI-Europe (Zwijndrecht, Belgium), respectively. VivoTrax™ (Resovist^®^ licensed for distribution in USA for pre-clinical MPI studies), was purchased from Magnetic Insight (Alameda, CA, USA). Polyhydroxyalkanoates was extracted from a bacterial strain Pseudomonas mendocina CH50 and hydrolyzed into Oligo-polyhydroxyalkanoates (10 kDa) by the group of Prof. Ipsita Roy. All the chemicals were used as received without any further purification. Milli-Q water (18.2 MΩ, filtered with filter pore size 0.22 µM) was filtered using Millipore distributed by Merck Life Science S.r.l. (Milan, Italy).

## 3. Methods

### 3.1. Synthesis of Core-Shell Iron Oxide Nanocubes

Mixed-phase core-shell FeO/Fe_3_O_4_ nanocubes (core-shell IONCs) with an average cube-edge length of 18 ± 2 nm were synthesized according to a previously published method [27]. Briefly, 5 mL of ODE, 1.98 g of OLAC, and 0.91 g of sodium oleate were mixed in a 50 mL three-neck glass flask. The flask was then connected to a Schlenk line through a condenser and degassed at 110 °C for ~1 h until no further bubbling was observed. Afterward, the reaction mixture was cooled to 60 °C by temporarily removing the heating mantle and then 400 μL (3 mmol) of Fe(CO)_5_, freshly dissolved in 1 mL of ODE in the glove box, was injected into the flask. The resulting yellowish mixture was heated to 320 °C within 25 min. The mixture first turned into dark orange and after 1 h of aging to a black color, indicating the nucleation of seed crystallites. From the time of the nucleation, the mixture was kept at 320 °C for an additional 90 min to grow the nanoparticles. The iron precursor injection and the growth step were carried out under a nitrogen atmosphere. After cooling the resulting black nanoparticle suspension to 80 °C, 40 mL of CHCl_3_ was added to prevent flocculation and facilitate the dispersion of the nanoparticles. After adding CHCl_3_, the sample was further cooled to room temperature (RT). The resulting nanoparticles were precipitated by adding a 1:2 methanol/acetone solution and collected by centrifugation at 6000 rpm for 10 min. The precipitated nanoparticles were dispersed in fresh CHCl_3_ and the addition of anti-solvents and centrifugation steps were repeated five more times to clean the nanoparticle sample. Finally, the isolated nanoparticles were dispersed in 40 mL CHCl_3_ by sonication and kept at 4 °C for use in the succeeding experiments.

### 3.2. Synthesis of Single-Phase Iron Oxide Nanocubes

Single-phase iron oxide nanocubes (single-phase IONCs, Fe_3_O_4_ composition) with an average cube-edge length of 16 ± 2 nm were prepared according to our previously published protocol [25,28]. A proportionate amount of iron (III) acetylacetonate (0.353 g) and decanoic acid (0.69 g) in a mixture of squalane (7 mL) and dibenzyl ether (18 mL) were degassed for 120 min at a temperature of 65 °C and then heated to 200 °C (3 °C/min) for 2.5 h. Next, the temperature was raised further to 310 °C (7 °C/min) and maintained at this refluxing temperature for 1 h. After cooling the reaction mixture, the nanocubes were precipitated by the addition of acetone, and further centrifugation with this washing step was repeated two times before collecting the final nanocube pellet, which was redispersed in CHCl_3_.

### 3.3. Fabrication of Anisomeric Nanoclusters Using Core-Shell IONCs as Building Blocks

The core-shell IONCs were clustered into organized assemblies according to a previously published method [24]. In a controlled manner, three different morphologies were fabricated, namely single nanocrystals (Si), dimers/trimer (Di/Tri), and 3D-Clusters, using a commercially available poly(styrene-co-maleic anhydride) polymer (PScMA) as the encapsulating agent. Briefly, a mixture of core-shell IONCs (0.22 mg_Fe_) and PScMA in THF (10 mL, 2.19 mM), under sonication at 10 °C, was destabilized by the addition of 1 mL of Milli-Q water (flow rate 0.5 mL/min), followed by a slow solvent evaporation step conducted at room temperature and under shaking at 100 rpm. The ratio of polymer chains per square nanometer of nanocube surface was exploited as the main parameter to control the cluster geometry. Henceforth, for obtaining, Si, Di/Tri, and 3D-Clusters, 12, 18, and 35.5 molecules of PScMA/nm^2^ were used, respectively. To phase transform the core-shell structure (FeO/Fe_3_O_4_) into a single phase Fe_3_O_4_ dominated crystal structure, the as-prepared assemblies once transferred into water, were thermally oxidized by exposure to a water bath set at 80 °C for a time of 48 h, to guarantee full conversion of the FeO/Fe_3_O_4_ mixed-phase to the desired single Fe_3_O_4_ crystal phase [24].

### 3.4. Fabrication of 2D-Clusters Using Single-Phase IONCs

The single-phase IONCs having an average cube-edge length of 16 ± 2 nm were assembled into water-soluble, two-dimensionally ordered magnetic clusters (2D-Clusters) by using a bacteria-derived polymer oligo-polyhydroxyalkanoate (oligo-PHA) ligand, according to a previously published protocol [25,29]. In this solution-phase self-assembly approach, a stable mixture of hydrophobic IONCs (28 µL, 2.22 mg_Fe_/mL in CHCl_3_) and oligo-PHA polymer (30 µL, 2 mM in CHCl_3_, 10 kDa) were re-dissolved in 200 µL of fresh THF, after evaporating the initial CHCl_3_ solvent and gradual destabilization by the dropwise addition of Milli-Q water (0.5 mL/min) under mild shaking at 420 rpm. The sample was magnetically collected from the THF suspension by exposure to a small permanent magnet (0.3 T). The same process was repeated 20 times to obtain a sufficient amount of materials needed for the succeeding MRI/MPI measurements.

### 3.5. Characterization of the IONC Cluster Assemblies

The mean hydrodynamic sizes of the synthesized nanoclusters were evaluated by using a commercial Zetasizer Nano ZS90 (Malvern, UK) dynamic light scattering (DLS) system equipped with a He-Ne laser (4.0 mW) of 633 nm and a photodiode detector. The cluster morphologies were further confirmed under transmission electron microscopy (TEM; JEOL JEM-1011, Tokyo, Japan) operated at 100 kV. TEM samples were prepared through drop-casting a few drops of the diluted cluster suspensions and subsequent drying of the samples on carbon-coated copper grids. Quantification of iron in each sample, which is necessary for the MPI and MRI performance comparison studies, was performed by using an inductively coupled plasma-atomic emission spectrometer (ICP-AES, iCAP 7600 DUO, Thermo, Bremen, Germany) after overnight digestion of the samples in aqua regia. Magnetic measurements were performed on a superconducting quantum interference device (SQUID) from Quantum Design-Europe (Danderyd, Sweden). The magnetization curves were recorded within the magnetic field of ± 70 kOe. Samples were prepared by dropping 50 µL of nanoparticle solution in water (ca. 1 g_Fe_/L) in a polycarbonate capsule filled with 50 mg of calcium sulfate dihydrate and the mixture was left to dry for 12 h. The magnetization curves were normalized to the iron concentration derived from the elemental analysis.

### 3.6. MPI Relaxometry Measurements

MPI analyses were performed in a custom made MPI relaxometry device [13,30]. Representative samples of Si, Di/Tri, and 3D-Clusters prepared from core-shell IONCs were analyzed as aqueous suspensions of 250 µL at a fixed iron concentration of 0.5 mg_Fe_/mL. The samples were sonicated before performing each MPI relaxometry measurement. In the case of the 2D-Clusters made with single-phase IONCs, the MPI analysis was made at a volume of 450 µL at 0.5 mg_Fe_/mL. All MPI studies were conducted under a magnetic excitation field strength and frequency of 20 mT and 16.8 kHz, respectively. The MPI signals generated were normalized to the MPI signal of a commercial MPI tracer (VivoTrax™).

### 3.7. Monitoring Changes in the MPI Signal of 2D-Clusters upon Exposure to Esterase Enzyme

To 450 µL aqueous suspension of the 2D-Clusters (0.5 mg_Fe_/mL), 23 µL of esterase enzyme (2 mg/mL) was added and the sample was incubated for 48 h at 37 °C, mimicking the biological environment. During the course of the enzymatic reaction, the MPI relaxation signals were recorded at several time points, starting from 0 h to 48 h. Moreover, the physical appearance of the sample at each time point was photographed for comparison.

### 3.8. MRI Relaxivity Measurements

The proton relaxation rates of the Si, Di/tri, 3D-Clusters, and 2D-Clusters samples were analyzed using a Minispec spectrometer (Bruker, Germany, mq60, (1.5 T)) [28,31]. The samples were diluted to obtain aqueous nanoparticle suspension series containing an iron concentration ranged from 0.009 mM to 0.6 mM. For the 2D- and 3D-Clusters, 0.1% of agarose aqueous solution was used as a diluting solvent rather than water. The relaxation constants *r*_1_ and *r*_2_ (longitudinal and transverse relaxivity, respectively) were evaluated from the linear slope of the relaxation time curves (1/*T*_2_ or *r*_2_ in mM^−1^ s^−1^) versus Fe concentration. The samples were sonicated for 30 s before performing each measurement. The relaxation of the 2D-Clusters after exposure to the esterase enzyme (10 µL, 2 mg/mL) and incubated at 37 °C was also recorded at well-defined time intervals (0, 0.25, 0.5, 1, 3, 6, 12, 24, and 48 h).

## 4. Results and Discussion

**Figure 1:**
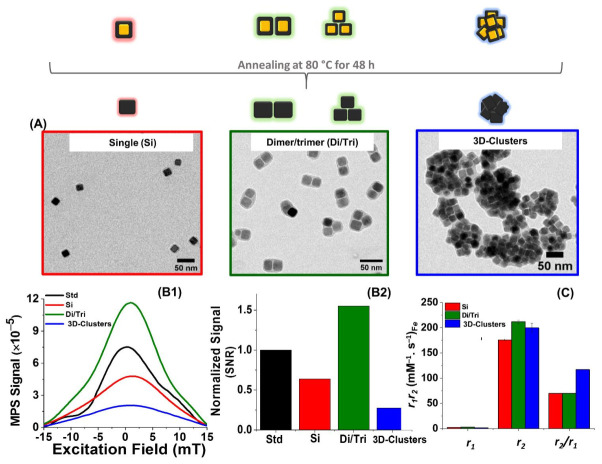
Scheme of the thermal annealing step performed on clusters to promote the transformation of core-shell nanocubes in the clusters to single Fe_3_O_4_ phase. (**A**) Representative TEM images of MNP assemblies made from core-shell IONCs and encapsulated within a PScMA polymer matrix having a single (Si) nanocube, a short-chain dimer/trimer (Di/Tri), and a 3D-Clusters configuration, respectively. Please note that the cluster samples were thermally annealed prior to TEM image collection to convert the core-shell IONCs into single-phase Fe_3_O_4_. (**B1**,**B2**) MPI analysis of the nanocube assemblies: (**B1**) shows the point spread function (PSF) obtained for each sample in comparison to the MPI signal of a commercial reference standard VivoTrax™ (Std) measured at 16 kHz and 20 mT, and (**B2**) displays the histograms of the corresponding signal to noise ratios (SNRs), which have been normalized to the signal of the reference standard. (**C**) Magnetic resonance relaxation rates of all the three structures measured in water upon exposure to a static magnetic field of 1.5 T.

To study the MPI and MRI performance of the different nanocube clusters, we first prepared a series of nanoclusters with well-defined geometries using the core-shell FeO/Fe_3_O_4_ nanocubes (18 ± 2 nm, Supplementary Figure S1), prepared following a thermal decomposition method [27]. Magnetic assemblies of the FeO/Fe_3_O_4_ nanocubes with different geometries were fabricated using a cumene terminated amphiphilic polymer, poly(styrene-*co*-maleic anhydride) (PScMA) as the encapsulating ligand, through a microemulsion based approach. Through the slow evaporation of THF in the THF/water solvent mixture containing the polymer and nanocubes, a change in solvent polarity occurs that drives the assembly formation [24]. By controlling the amount of the ratio polymer molecules/nm^2^ of nanocube surface, it was possible to tune the morphology of the nanocube assemblies into single nanocrystals (Si), short-chains of two or three nanocubes, named dimer/trimer (Di/Tri), and spherical cluster assemblies (3D-clusters). It is worthy to mention that this clustering protocol was feasible only for non-interacting core-shell IONCs (Supplementary Figure S1) and does not work if single-phase IONCs of Fe_3_O_4_ composition were used as starting material (Supplementary Figure S2). This is due to the strong magnetic dipolar interactions occurring between single-phase Fe_3_O_4_ IONCs during the solvent evaporation process that led to nanoparticle aggregation, even if the chosen nanocubes were as small as 13 ± 2 nm in cube-edge length. In addition, when using the core-shell FeO/Fe_3_O_4_ nanocubes, the encapsulation was just straightforward due to considerably weaker magnetic interactions occurring between the core-shell nanocubes having a large core of FeO, a paramagnetic and low interacting iron oxide phase. In order to obtain a Fe_3_O_4_ nanocube assembly from the core-shell IONCs, a thermal annealing process was required. To the clusters suspended in water, thermal exposure to 80 °C for 48 h induced a slow phase transformation of the magnetically inactive wüstite phase (FeO) to the highly magnetic magnetite (Fe_3_O_4_) phase without compromising the polymer coating and the structure of the assemblies [24]. As shown in Figure 1A, the TEM images of clusters with Si, Di/Tri, and 3D-Clusters after the thermal annealing process showed well-defined morphologies of the different geometrical assemblies. The aqueous colloidal stability of the different clusters was verified from DLS measurements. The mean hydrodynamic diameters (*D*_H_) for the Si, Di/Tri, and 3D-Clusters were evaluated as 55 ± 4 nm, 66 ± 4 nm, and 173 ± 5 nm, respectively, with polydispersity index values of less than 0.1 indicating narrow size distributions (Supplementary Figure S3).

To investigate the MPI performance of the different magnetic assemblies, MPI relaxometry experiments were performed using Case Western Reserve University’s custom x-space magnetic particle relaxometer [13,30]. MPI relaxometry measurements were obtained for the Si, Di/Tri, and 3D-Clusters samples (0.5 mg/mL of Fe in 250 μL of water) under sinusoidal magnetic field excitation at 16.8 kHz and 20 mT. The measured MPI signals, including both PSF and signal to noise ratios (SNRs) of the samples, were compared and normalized to a commercial MPI tracer (Std, VivoTrax™), which is licensed for distribution in the US as a pre-clinical MPI tracer (Figure 1B1,2). Among the different magnetic assemblies investigated, the short-chain like assemblies of the Di/Tri sample exhibited the highest MPI signal compared to the Si nanocubes, 3D-clusters, and commercial Std. The corresponding SNRs show that the short-chain Di/Tri sample has a 57% greater MPI response than the Si, and 85% better signal than the 3D-clusters respectively, and interestingly 36% higher than the MPI tracer VivoTrax™.

The unique enhancement in MPI signal can be likely attributed to the strong uniaxial magnetic dipolar coupling occurring in the chain assembly [24]. This observation is in strong agreement with our previous work on studying MH heat performance of the assembly series, which revealed the same trend of performance in terms of the measured specific absorption rate (SAR) values [24]. In our previous published studies, we reported that the Di/Tri sample showed the best heating efficiency due to the linear magnetic dipolar coupling in the system that affects the overall magnetic property of the nanocube assembly [24]. These finding for the Di/Tri sample is also in good agreement with the recent MPI relaxometry study of Müssig et al. on anisotropic rod-like microscale assemblies of MNPs [32]. The authors reported, the preparation under a permanent magnet (0.12 T) of meso/microscale rod-like assemblies of multiple superparamagnetic nanoparticles of an average size of 10 nm coated by a silica shell, which exhibited a 14-fold improvement in MPI signal, in comparison to isotropic aggregates (150 nm to 8 µm assemblies produced in the absence of a magnetic field gradient) and individual particles. This unique enhancement was claimed to depend on the chain-like anisotropic assembling of the MNPs rather than the intrinsic property of individual nanoparticles used as building blocks [32]. Moreover, the reduced MPI signal of the 3D-Clusters we observed, is likely attributed to the random magnetic dipole interactions in the centrosymmetric cluster system [33]. Indeed in such 3D conformation, the consequent demagnetization resulting from random orientation and destructive dipolar interactions of the nanocubes within the 3D-Clusters [34,35] may compromise its response to AC magnetic fields, thus lowering the MPI signal. Moreover, the impact of limited Brownian motion may also affect the MPI signal as recently hypothesized by Wu et al. [14]. In their study, the authors investigated streptavidin-specific clustering of biotin functionalized iron oxide nanoparticles and correlated the change in the streptavidin-biotin interactions to the variation in MPI signal as a function of the increase in streptavidin concentration. The authors found that the addition of streptavidin resulted in the formation of large nanoparticle aggregates with large hydrodynamic sizes, which inhibited the MNP rotational freedom and led to a decrease in the Brownian motion that has a direct effect of lowering the harmonic signal amplitudes [14].

Aside from the MPI capabilities, the nanocube assemblies were also found to exhibit remarkable MRI *T*_2_ relaxation rates (*r*_2_) corresponding to 176 mM^−1^ s^−1^ for the Si nanocubes, 212 mM^−1^ s^−1^ for the Di/Tri sample, and 200 mM^−1^ s^−1^ for the 3D-Clusters, upon measuring their magnetic relaxation rates at 1.5 T static magnetic field exposure (Figure 1C and Figure S4). Moreover, for all the assemblies, the *T*_2_ relaxation values were generally higher than most commercial MRI contrast agents [36]. Although the *r*_2_ of the Di/Tri sample is higher than those of the Si nanocubes and 3D-Clusters, the corresponding *r*_2_/*r*_1_ ratio of the 3D-Clusters (117) is higher than that of the Si and Di/Tri samples (*r*_2_/*r*_1_ = 70 for both Si nanocubes and Di/Tri sample), which is consistent to previous findings reported on similar morphologies but composed of different types of iron oxide nanoparticles [28,37].

**Figure 2:**
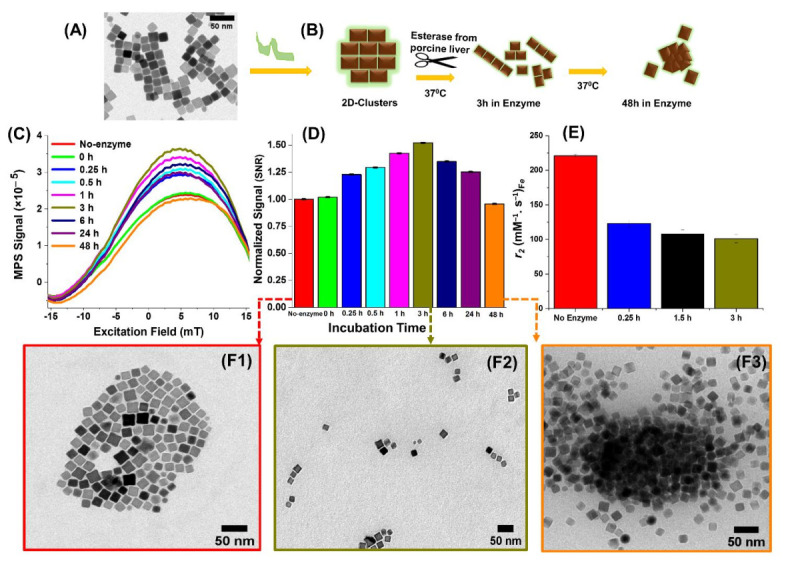
(**A**) Representative TEM image of iron oxide nanocubes used in the fabrication of the 2D-Clusters. (**B**) Schematic representation of the enzymatic incubation study of the 2D-Clusters in response to an esterase enzyme. (**C**) The progression of the PSF of the 2D-Clusters upon exposure to esterase at different time points for a total incubation period of up to 48 h, analyzed at 16 kHz and 20 mT, and (**D**) the histogram of the corresponding SNRs. (**E**) Magnetic resonance relaxation rates of the 2D-Clusters before and after exposure to the enzyme measured in water after exposure to 1.5 T of a static magnetic field. (**F1**–**F3**) TEM images of the initial 2D-Clusters (**F1**) with no enzyme treatment and after a 3 h (**F2**) and 48 h incubation (**F3**) period with the esterase enzyme.

Following the investigation of the MPI/MRI performance of the different magnetic nanocube assemblies, we further implemented a magnetic assembly that is responsive to an esterase enzyme. For this part of our study, iron oxide nanocubes (IONCs, 16 ± 2 nm, Figure 2A) of single-phase Fe_3_O_4_ composition were encapsulated into 2D-Clusters using a different approach than the one used for the core-shell nanocube system. Here, we utilized an enzymatically cleavable polymer, polyhydroxyalkanoate (PHA), for the 2D-Clusters formation [25]. This polymer was extracted from a bacterial source, *Pseudomonas mendocina* CH50 [29], and was subsequently hydrolyzed into short oligomeric forms of 10 kDa (oligo-PHA) [25]. To prepare the enzyme responsive 2D-Clusters, a mixture of IONCs together with oligo-PHA in THF was destabilized by the dropwise addition of water. The amphiphilicity and polymer linearity [38] in synergy to other reaction parameters (e.g., mild shaking speed and slow anti-solvent addition), directed the nanocube assembly into controlled 2D-Clusters [25] possessing good colloidal stability in water (Figure 2 and Supplementary Figure S5 for the *D*_H_ value in water).

Since the polymer used to fabricate the 2D-Clusters is sensitive to esterase [26,39], here, the change in MPI/MRI signals was investigated over time, upon exposure to porcine liver esterase at 37 °C, using MPI and MRI relaxometry (Figure 2B, scheme). Remarkably, following the addition of the enzyme, the MPI signal gradually increased with the incubation time and reached maximum values, both in terms of PSF and SNR signals, after an incubation time of 3 h (Figure 2C,D). The relatively high MPI signal at 3 h can be attributed to the structural disassembling of the initial 2D-Clusters (Figure 2F1) into shorter chain-like fragments as confirmed by TEM images (Figure 2F2). This enhancement is consistent with the MPI signal observed for the Di/Tri (Figure 1) sample, although the nanocubes used as building blocks for the two types of arrangements were prepared by different synthetic methods and therefore provide MPI signals significant different with each other. As reported by others [23,25,40], it is likely that the uniaxial dipolar coupling of the magnetic easy axis of nanoparticles inside the chains, improves the magnetization along the length of the chain enabling the sample to respond well to the AC magnetic field excitation during MPI measurement. However, the MPI signals after 24 h and 48 h of incubation period, dropped beyond the maximum values at 3 h, whereby TEM confirmed the presence of precipitate formation (Figure 2F3) and large aggregates (Supplementary Figure S6), which likely led to reduced Brownian relaxation and diminished the MPI response as it happened for the 3D-Clusters. It is also worth to mention that if the same Fe_3_O_4_ IONCs batch used for the 2D-Clusters were dispersed in water as single nanoparticles, they possess MPI signal that is better than that of the commercial Std or the same nanocubes arranged in a 2D or a 3D configuration (Supplementary Figure S7). These results may be likely related to the magnetization saturation values of the IONCs and the 2D-Clusters. The single nanocubes and the 2D-Clusters exhibit typical superparamagnetic behavior at 298 K (Figure. S8) with saturation magnetization (*M*_s_) value of 105 emu/g_Fe_ for individually coated IONCs and a slightly lower value of 95 emu/g_Fe_ for the 2D-Clusters. These *M*_s_ values are also comparable (slightly higher) to the *M*_s_ values at room temperature previously measured for the Si, Di/Tri, and 3D-Clusters and after phase transformation from core-shell to the Fe_3_O_4_ single phase [23].

Aside from the structurally sensitive MPI signal, the 2D-Clusters under an enzyme-free condition also showed a good *T*_2_ relaxation rate at 1.5 T static field, with an absolute *r*_2_ value of 221 mM^−1^ s^−1^ (no enzyme, red bar in Figure 2E). However, the *r*_2_ value significantly decreased upon exposure to the enzyme already after a short incubation period exposure (123 mM^−1^ s^−1^ at 0.25 h) reaching 101 mM^−1^ s^−1^ at 3 h (Figure 2E and Supplementary Figure S9). These findings can be associated to the change of the assembly configuration and possible variations in water exposure of the nanocubes during the disassembly process. However, given that the *r*_2_ signal drastically decreased to half of its value from the 2D-Clusters configuration to the chain-like fragments formed at 3 h of enzyme incubation, the enzymatic digestion process that led to the formation of the chain-like assemblies can also be followed by monitoring the MRI signal.

## 5. Conclusions

In summary, we have measured the MPI and MRI relaxation properties of different magnetic nanostructure assemblies fabricated from iron oxide-based nanocubes. Owing to the improved magnetic coupling of the nanocubes, our chain-like assembled samples (Di/Tri samples as well as the chains derived by the enzymatic degradation of the 2D-Clusters) exhibited the best MPI performance compared to VivoTrax™, a commercial MPI tracer. Furthermore, the Di/Tri samples generated a better MPI signal than the centrosymmetric 3D-Clusters and single coated nanocubes prepared using the same type of polymer and core-shell nanocube particles. Moreover, the disassembled chains derived from the initial PHA-coated 2D-Clusters, fabricated upon enzymatic digestion, were able to provide a significantly enhanced MPI signal, which is reflected in the change in MPI performance upon exposure of the 2D-Clusters to esterase (Figure 2). This latter result may suggest the possibility to track the disassembly process of 2D-Clusters in vivo following the MPI signal evolution. In addition, all these nanostructures prove to have great potential as *T*_2_ MRI contrast agents. In the case of the transformation from 2D-Clusters to chain-like fragments, the drastic reduction in *r*_2_ signal can also be exploited as a MRI signal fingerprint for following the enzymatic degradation action. Therefore, we may exploit the MPI signal enhancement and the MRI signal reduction on 2D-Clusters as a dual way to verify and detect the esterase enzymatic action as a tumor microenvironment marker. Overall, the magnetic imaging property findings on our iron oxide based nanocube clusters, together with their previously demonstrated thermo-therapeutic properties [24,25], suggest the possibility to exploit these nanoplatforms for dual MPI/MRI diagnostic imaging tools coupled to their use as therapeutic agents in magnetic hyperthermia.

## Data Availability

Data sharing not applicable.

## Supplementary Materials

The following are available online at https://www.mdpi.com/2079-4991/11/1/62/s1, Figure S1: TEM image of the core-shell IONCs (FeO/Fe3O4 composition); Figure S2: Di/Tri production with 13 nm single phase IONCs with initial Fe3O4 composition; Figure S3: DLS data of Si, Di/Tri, 3D-Clusters; Figure S4: Linear magnetic relaxation slopes of Si, Di/Tri, and 3D-Clusters; Figure S5: DLS peaks of 2D-Clusters; Figure S6: Photographs of 2D-Clusters suspension at different time points of incubation with esterase enzyme at 37 °C; Figure S7: MPI measurements of single-core IONCs in an isolated and 3D configuration in comparison to the 2D-Clusters and the Std; Figure S8: Magnetic characterization of the single IONCs in comparison to 2D-Clusters; Figure S9: Linear magnetic relaxation slopes of 2D-Clusters at different time points of incubation with esterase.
