# Supplementary material for: Uncovering the Magnetic Particle Imaging and Magnetic Resonance Imaging Features of Iron Oxide Nanocube Clusters
Source: Nanomaterials (Basel). 2020 Dec 29;11(1):62. doi: 10.3390/nano11010062 (PMC7824301; doi:10.3390/nano11010062)
Supplement: Supplementary file 1 [file nanomaterials-11-00062-s001.pdf]

Electronic Supporting information

Uncovering the Magnetic Particle Imaging and Magnetic Resonance Imaging Features of Iron Oxide Nanocube Clusters

Sahitya Kumar Avugadda,[1] Sameera Wickramasinghe,[2] Dina Niculaes,[1] Minseon Ju,[2] Aidin Lak,[1] Niccolò Silvestri,[1] Simone Nitti,[1] Ipsita Roy,[3] Anna Cristina S. Samia*[2] and Teresa Pellegrino*[1]

[1] Istituto Italiano di Tecnologia, via Morego 30, 16163 Genoa, Italy.
[2] Department of Chemistry, Case Western Reserve University, 10900 Euclid Avenue, Cleveland, Ohio 44106, United States.
[3] Department of Materials Science and Engineering, University of Sheffield, Sheffield, S10 2TN

* Correspondence: Teresa.pellegrino@iit.it; anna.samia@case.edu.

Received: date; Accepted: date; Published: date

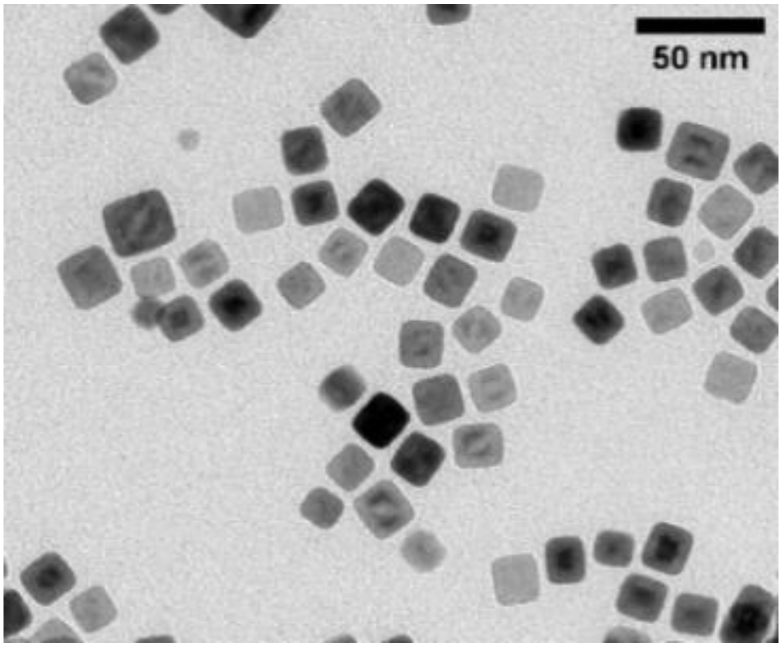

Figure S1. Representative TEM image the core-shell (FeO/Fe_3O_4) iron oxide nanocubes of edge length 18 ± 2 nm in $CHCl_3$ chosen for the preparation of the single coated (Si), dimers and trimers(Di/Tri), and centrosymmetric clusters (3D-Clusters).

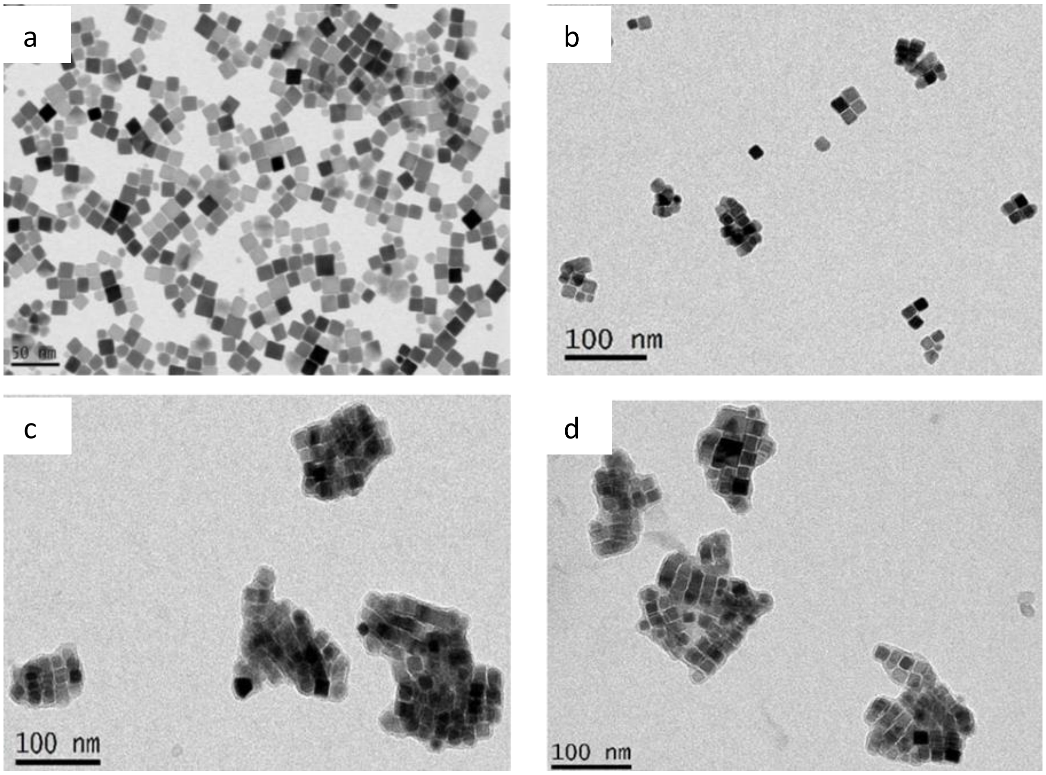

Figure S2. Control experiment: attempt to produce Dimeric and trimeric clusters using poly(styrene-co-maleic anhydride) amphiphilic polymer (PS*c*MA, 1600 Da) and single-phase Fe_3O_4 nanocubes of cube edge of 13 ± 2 nm instead of core-shell FeO/Fe_3O_4 nanocubes. The protocol to get short chain from single-phase Fe_3O_4 is not as controlled as for the core/shell FeO/Fe_3O_4 NPs probably due to stronger nanocube interparticle magnetic interactions than in the adapted core-shell FeO/Fe_3O_4 nanocube system. a) As-synthesized Fe_3O_4 iron oxide nanocubes, the reaction of clustering at a ratio of PS*c*MA molecules/nm^2 of b) 12, c) 18, and d) 35.5, respectively. The reactions performed under these conditions produced deformed structures with poor water dispersion and a low cluster yield. These experiments indicate the importance to choose core-shell FeO/Fe_3O_4 NPs for the clustering protocol.

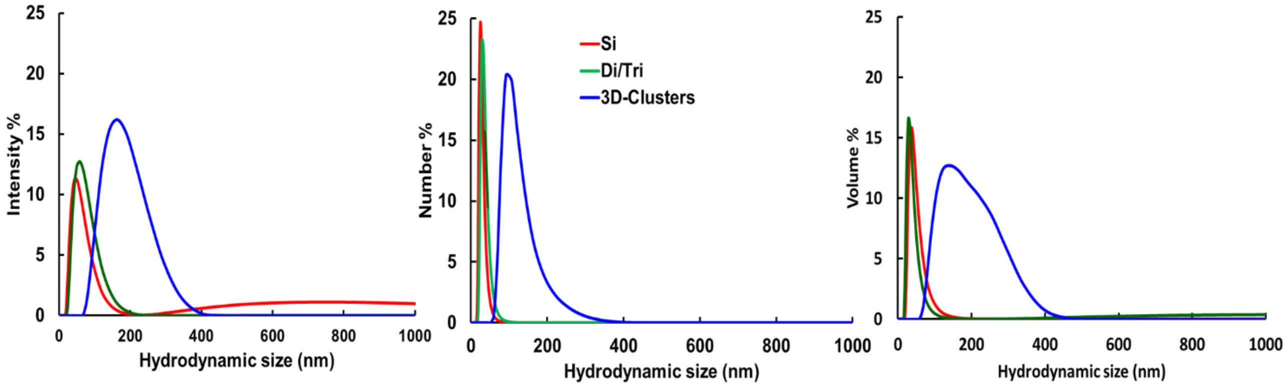

Sample	H.Size (nm)			PDI
	Number %	Intensity %	Volume %	
Si	28±2	55±4	37±1	0.275
Di/Tri	34±3	66±4	45±2	0.2
3D-Clusters	116±3	173±5	172±6	0.095

Figure S3: The mean hydrodynamic size (D_H) curves and polydispersity index (PDI) values for Si, Di/Tri, and 3D-Clusters in water and after the thermal annealing process at 80 °C.

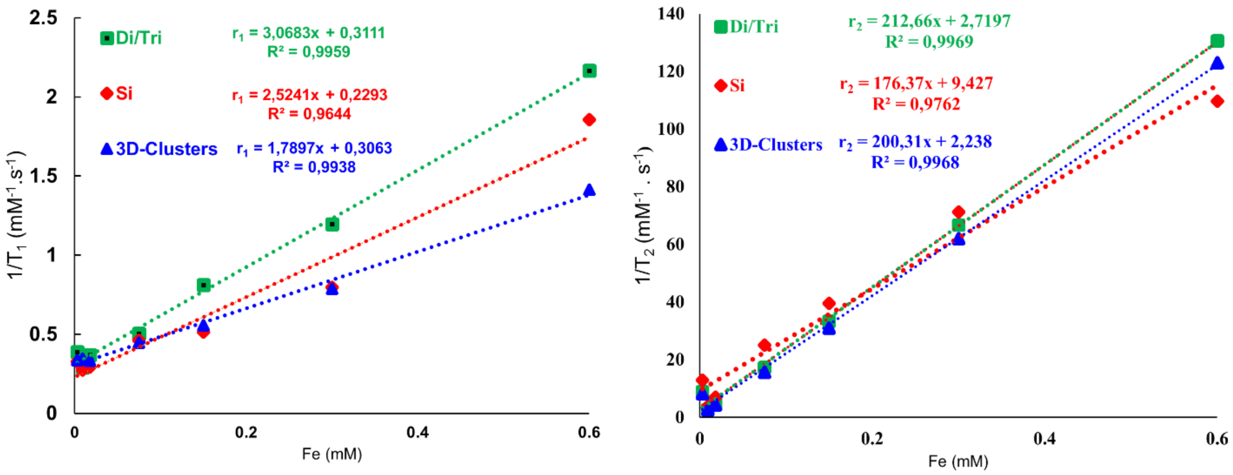

Figure S4. Calibration curves of r_1 ($1/T_1$) and r_2 ($1/T_2$) versus iron concentration with the corresponding slope values (relaxation times) and corresponding coefficients of determination (R^2) obtained for Si, Di/Tri, and 3D-Clusters, respectively.

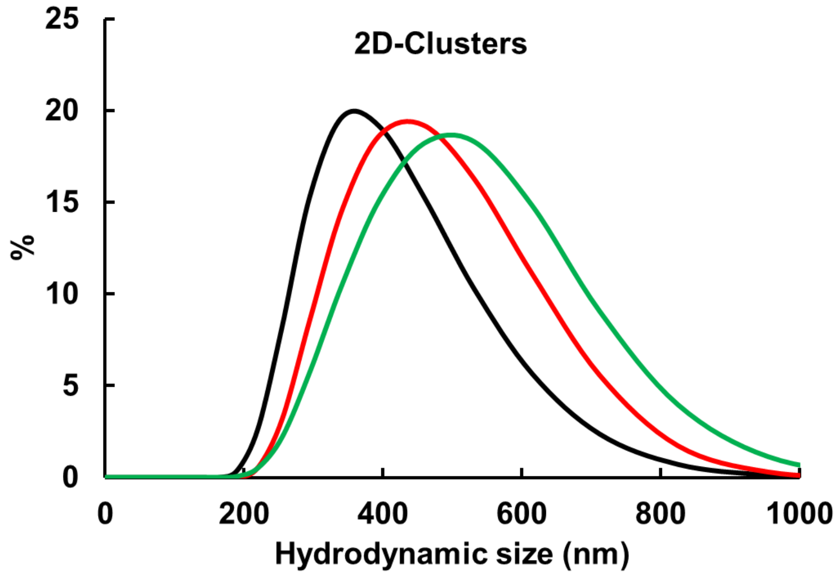

Figure S5. The mean hydrodynamic size (D_H) peaks of 2D-Clusters estimated based on the number percent (black curve), volume percent (red curve), and intensity percent (green curve) of sample dispersed in water.

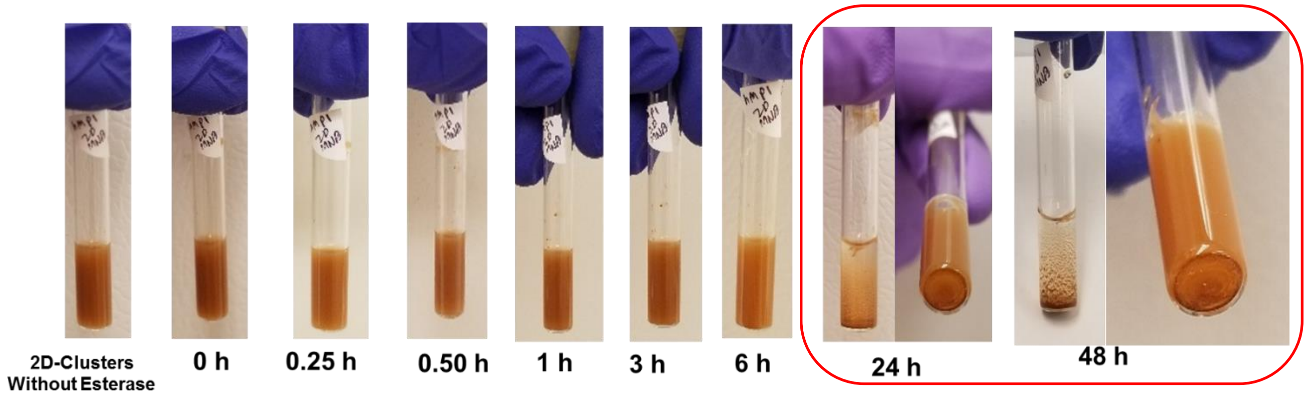

Figure S6. Photographs showing the appearance of 2D-Clusters suspension at different time points during the incubation with esterase enzyme at 37 °C. The images highlighted in the red panel correspond to 24 h and 48 h incubation time where a clear sign of sample precipitation is shown. This is not the case for samples up to 6 h of incubation.

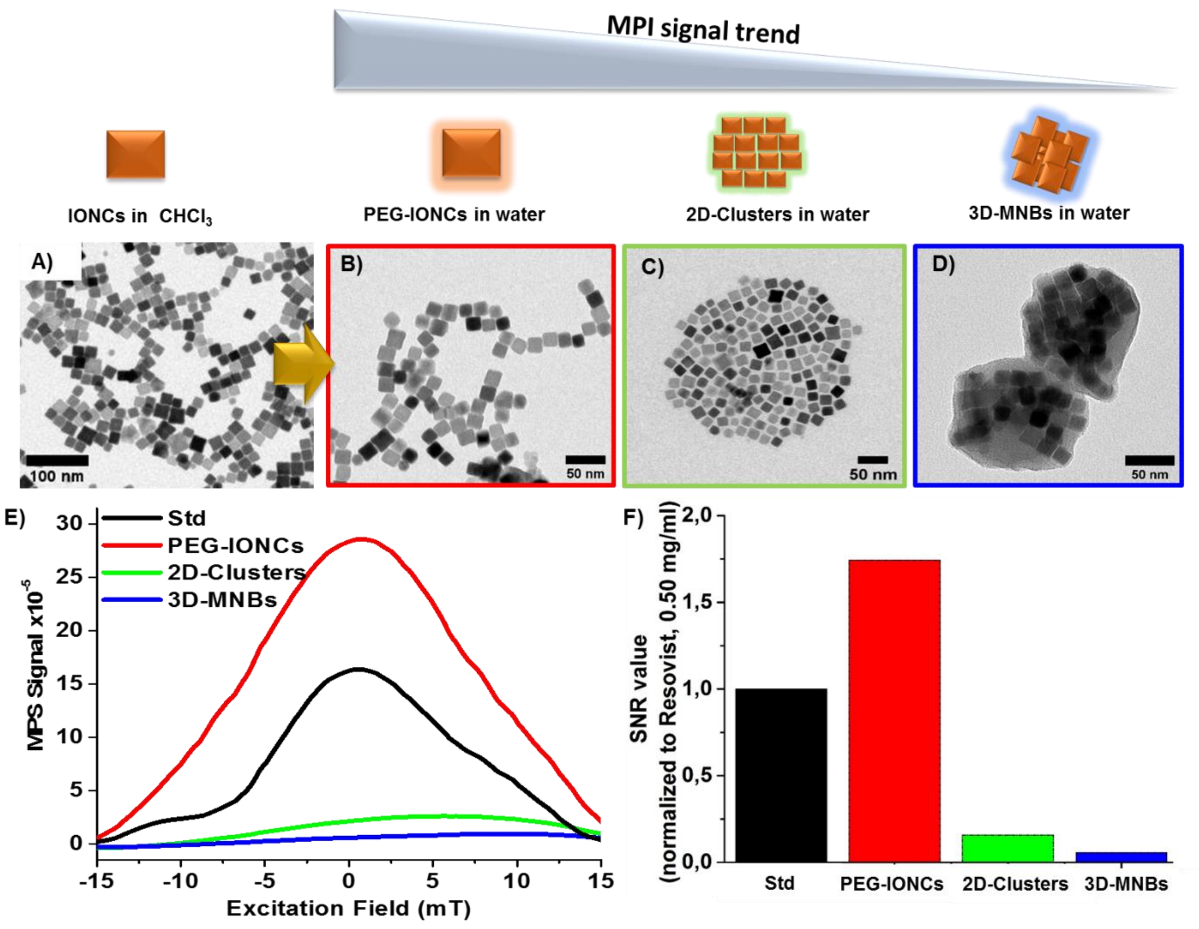

Figure S7. TEM images of the: A) as-synthesized single-phase iron oxide nanocubes (IONCs) of edge length 16 ± 2 nm dispersed in $CHCl_3$, B) individual iron oxide nanocubes coated with the nitrodopamine-PEG carboxylic-terminated ligand (PEG-coated IONCs) according to a published protocol [1] and deposited from a water dispersion, C) 2D-Clusters obtained when using oligo-polyhydroxyalkanoate polymer, and D) Three-dimensional magnetic clusters (3D-MNBs) obtained when using poly(maleic anhydride-alt-1-octadecene) (30-50 kDa) polymer following a published protocol [1]. E) Point spread function (PSF) of all the structures in comparison to commercially available reference, VivoTrax™ (Std) used as a benchmark standard, measured at 16 kHz and 20 mT, and the histograms of the corresponding signal to noise ratios (SNRs) of the same samples.

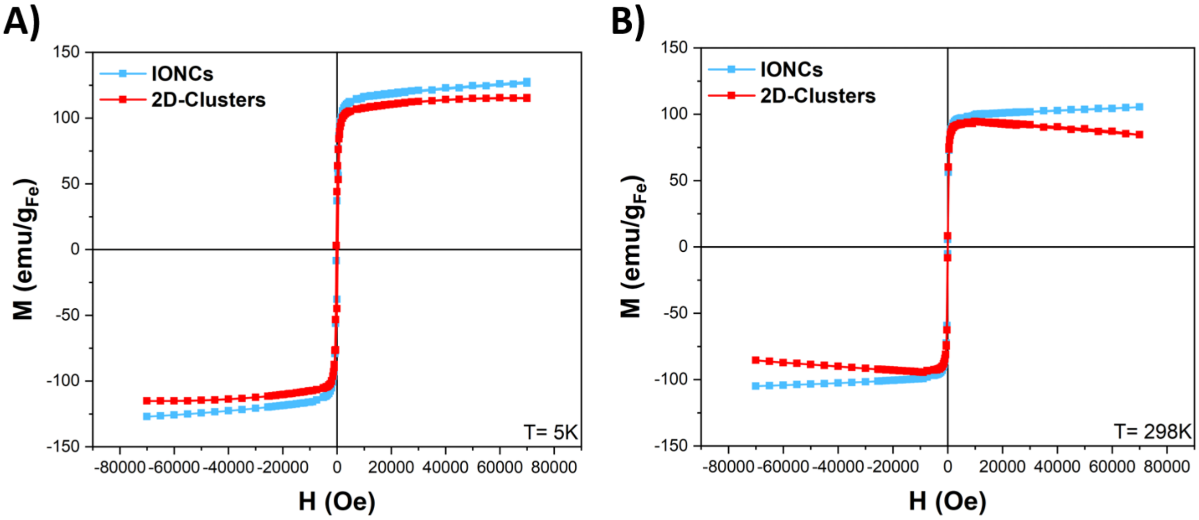

Figure S8. M-H magnetization curves of single coated IONCs (blu lines) and 2D-Clusters (red lines) were measured at 5 k (**A**) and room temperature (**B**). At room temperature, the diamagnetic contribution of the polymer on the 2D-Clusters is quite evident by the negative deflection of the red curve at increasing H values. For these measurements, the iron oxide nanocubes were transferred in water using tetramethylammonium hydroxide ligands accordingly to a well-established published protocol [2].

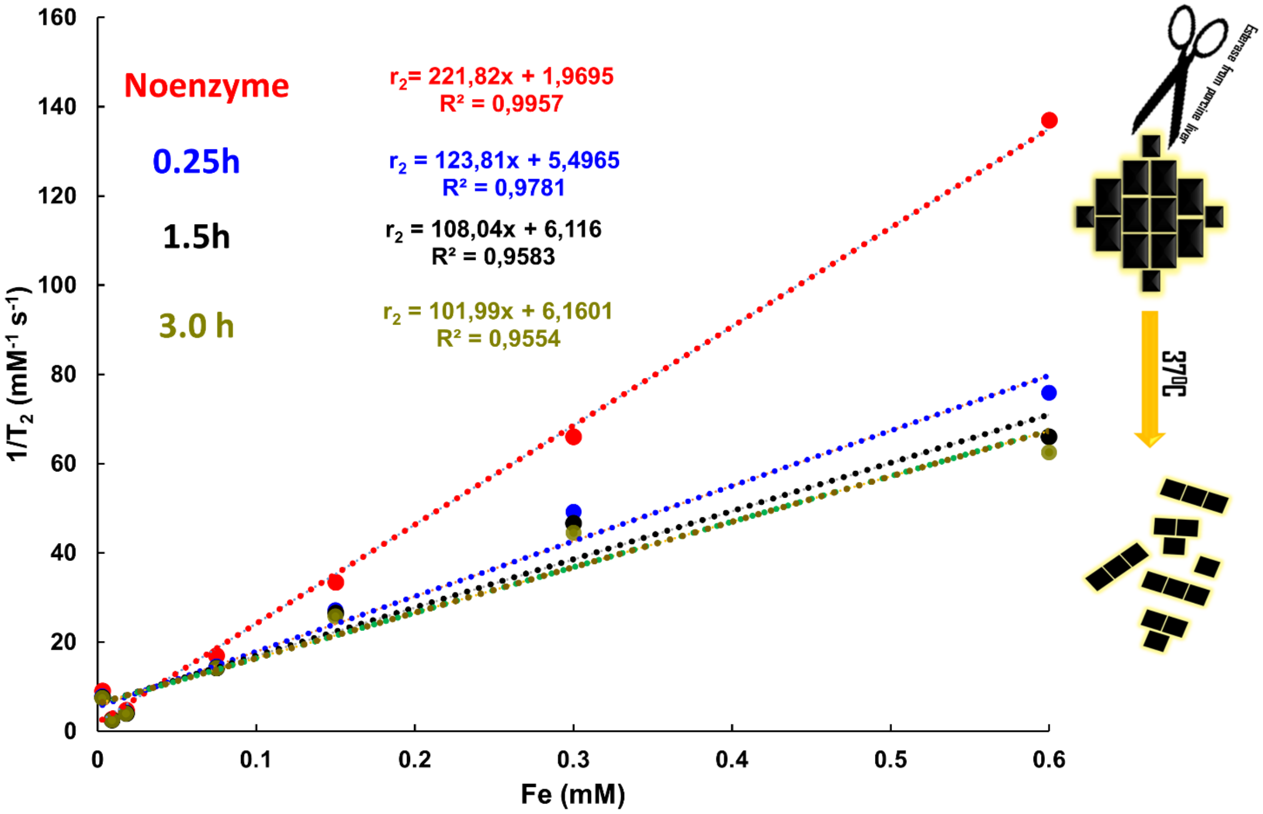

Figure S9. Calibration curves of $r_2 = 1/T_2$ versus iron concentration at different incubation time points with esterase at 37 °C, and the corresponding slope values (r_2 relaxation times) and coefficients of determination (R_2) of the linear curves obtained for the 2D-Clusters after 0.25, 1.5 and 3 h esterase incubation time periods, respectively.

References

1. Avugadda, S.K.; Materia, M.E.; Nigmatullin, R.; Cabrera, D.; Marotta, R.; Cabada, T.F.; Marcello, E.; Nitti, S.; Artés-Ibañez, E.J.; Basnett, P. Esterase-cleavable 2D assemblies of magnetic iron oxide nanocubes: exploiting enzymatic polymer disassembling to improve magnetic hyperthermia heat losses. *Chem. Mater.* **2019**, *31*, 5450-5463.

2. Guardia, P.; Pérez, N.; Labarta, A.; Batlle, X. Controlled synthesis of iron oxide nanoparticles over a wide size range. *Langm*uir **20**10, *26*, 5843-5847.
